# Impact of tissue kinetic heterogeneity on PET quantification: case study with the L-[1-^11^C]leucine PET method for cerebral protein synthesis rates

**DOI:** 10.1038/s41598-017-18890-x

**Published:** 2018-01-17

**Authors:** Mattia Veronese, Alessandra Bertoldo, Giampaolo Tomasi, Carolyn Beebe Smith, Kathleen C. Schmidt

**Affiliations:** 1 Section on Neuroadaptation & Protein Metabolism, National Institute of Mental Health, Bethesda, Maryland USA; 2 Department of Neuroimaging, IoPPN, King’s college London, London, UK; 3 Department of Information Engineering, University of Padova, Padova, Italy; 4 Padua Neuroscience Center, University of Padova, Padova, Italy

## Abstract

Functional quantification with PET is generally based on modeling that assumes tissue regions are kinetically homogeneous. Even in regions sufficiently small to approach homogeneity, spillover due to resolution limitations of PET scanners may introduce heterogeneous kinetics into measured data. Herein we consider effects of kinetic heterogeneity at the smallest volume accessible, the single image voxel. We used L-[1-^11^C]leucine PET and compared rates of cerebral protein synthesis (rCPS) estimated voxelwise with methods that do (Spectral Analysis Iterative Filter, SAIF) and do not (Basis Function Method, BFM) allow for kinetic heterogeneity. In high resolution PET data with good counting statistics BFM produced estimates of rCPS comparable to SAIF, but at lower computational cost; thus the simpler, less costly method can be applied. With poorer counting statistics (lower injected radiotracer doses), BFM estimates were more biased. In data smoothed to simulate lower resolution PET, BFM produced estimates of rCPS 9–14% higher than SAIF, overestimation consistent with applying a homogeneous tissue model to kinetically heterogeneous data. Hence with lower resolution data it is necessary to account for kinetic heterogeneity in the analysis. Kinetic heterogeneity may impact analyses of other tracers and scanning protocols differently; assessments should be made on a case by case basis.

## Introduction

One of the great strengths of positron emission tomography (PET) is that it can be used to provide quantitative measurements of physiological and biochemical processes. Functional quantification with PET is generally based on kinetic modeling approaches that relate a particular biological process of interest to measurements of activity in blood and tissue following administration of a radiolabeled tracer^1,2^. Kinetic models used in PET are necessarily simplified representations of tissue processes, and one of the simplifying assumptions frequently made is that the tissue volume being analyzed is kinetically homogeneous, i.e., rates of blood flow, delivery and efflux of tracer to/from tissue, metabolism, and incorporation into labeled products do not vary within the tissue region examined. In brain these assumptions are difficult to meet. At spatial resolutions approximately an order of magnitude higher than PET, such as achieved in autoradiographic studies, one clearly sees heterogeneity of rates of blood flow, glucose metabolism, and protein synthesis across the brain^3^. The rates of these processes not only differ between gray and white matter, but they can also vary considerably within gray matter structures themselves, as for example in the cortical layers^4,5^. At the relatively lower spatial resolution of PET scanning, therefore, activities measured in brain can be expected to originate from kinetically heterogeneous mixtures of tissue. Application of kinetic models designed for homogeneous tissues to heterogeneous tissues leads to errors in estimated rates of cerebral blood flow and glucose metabolism, as well as to errors in estimates of receptor binding parameters^6–9^.

Two approaches are in current use to reduce the effects of heterogeneity on estimation of kinetic model parameters from which rates of the process of interest are determined: (1) reducing the volume of tissue in which activity is analyzed, and (2) utilizing an analysis approach that does not depend on the assumption of kinetic homogeneity. There is a limit with PET data of how small a volume of tissue can be examined, as the smallest unit of available data is the single image voxel. But even if the voxel size could be made arbitrarily small (even lower than neuroimaging standards of 1–2 mm in size) and possibly contain only kinetically homogeneous tissue, its measured activity will be affected by the resolution limit of the technique (a positron travels an average 1–3 mm before annihilation)^10,11^, the resolution limit of the PET scanner^12^ and the partial volume effect, a limiting characteristic of all tomographic imaging systems^13–15^.

In the present study we investigate the effect of tissue kinetic heterogeneity when PET quantification is performed at the voxel level, with the final aim of understanding whether the assumption of kinetic homogeneity within a voxel is applicable or not. In particular we are interested in (1) quantifying the degree of kinetic heterogeneity for a given PET tracer and (2) assessing the impact of PET scanner resolution and injected tracer dose on this problem.

We utilize data from L-[1-^11^C]leucine PET studies for quantitative measurement of rates of cerebral protein synthesis (rCPS) in human subjects^16–19^ to explore these questions. Although the specific conclusions for this tracer may differ from those of other tracers and other scanning conditions, the considerations and the techniques for examining them can be extended to all PET tracers in brain and non-brain tissues.

## Materials and Methods

### Quantification of rCPS with L-[1-^11^C]leucine

Two approaches have been validated for quantification of rCPS at the voxel level. They depend on the assumptions used to model the behavior of L-[1−^11^C]leucine in brain, specifically whether the tissue is modeled as kinetically homogeneous or kinetically heterogeneous.

**Figure 1:**
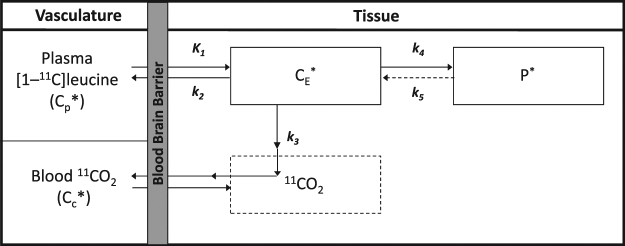
Homogeneous Tissue Model for L-[1-^11^C]Leucine PET method. In the homogeneous tissue model^16^ *K*_1_ and *k*_2_ are the rate constants for transport of leucine from plasma to tissue and back, respectively. *k*_3_ is the rate constant for the first two steps in leucine catabolism, transamination and decarboxylation, which yields ^11^CO_2_. *k*_4_ and *k*_5_ are the rate constants for leucine incorporation into protein and for the release of free leucine from proteolysis, respectively. Because of the long half-life of protein in brain^37^, it is assumed that there is no significant breakdown of labeled product (P*) during the experimental interval, i.e., *k*_5_P* ~ 0. Under the assumptions of negligible fixation of ^11^CO_2_ during the experimental period^38,39^ and rapid equilibration of ^11^CO_2_ between brain and blood^38^, the model reduces to two tissue compartments (*C*_E_*, which represents labeled leucine in the tissue, and P*) plus the ^11^CO_2_ compartment in which the concentration is assumed to be known. Total concentration of ^11^C in the field of view of the PET camera (*C*_T_^*^) is given by: ${C}_{T}^{\ast }(t)=(1-{V}_{b})\,[{C}_{E}^{\ast }(t)+\,{P}^{\ast }(t)+\,{V}_{D}{C}_{C}^{\ast }(t)]+\,{V}_{b}{C}_{b}^{\ast }(t)$where *V*_*b*_ is the fraction of the volume occupied by blood, *C*_b_^*^ is the total ^11^C concentration in whole blood, *C*_C_* is the concentration of ^11^CO_2_ in whole blood, and *V*_*D*_ is the blood:brain equilibrium volume of distribution of ^11^CO_2_. In term of the kinetic model rates constants we have: $$\begin{array}{c}{C}_{T}^{\ast }(t)=\,[(1-\,{V}_{b})\,\frac{{K}_{1}{k}_{4}}{{k}_{2}+{k}_{3}+{k}_{4}}]{\int }_{0}^{t}{C}_{p}^{\ast }(\tau )d\tau \\ \quad \quad \quad \quad +\,[(1-\,{V}_{b})\,\frac{{K}_{1}\,({k}_{2}+{k}_{3})}{{k}_{2}+{k}_{3}+{k}_{4}}]{\int }_{0}^{t}{C}_{p}^{\ast }(\tau ){e}^{-\beta (t-\tau )}d\tau \\ \quad \quad \quad \quad +\,{V}_{b}[{C}_{b}^{\ast }(t)-{V}_{D}{C}_{c}^{\ast }(t)]+\,{V}_{D}{C}_{c}^{\ast }(t)\end{array}$$ where *β* = *k*_2_ + *k*_3_ + *k*_4_. The model used to describe labeled leucine holds also for unlabeled leucine, except that unlabeled protein is assumed to be in steady state, and its steady state breakdown to amino acids including leucine is greater than zero, i.e., *k*_5_*P* > 0. Assuming no isotope effect, the rate constants are identical for unlabeled and labeled leucine.

The homogeneous tissue kinetic model^16^ is shown in Fig. 1. The parameters of the model are *K*_1_, the rate constant for transport of leucine from plasma to tissue; *k*_2_ + *k*_3_, the sum of the rate constant for transport of leucine from tissue to plasma plus the rate constant for the first two steps in leucine catabolism; *k*_4_, the rate constant for leucine incorporation into protein; and *V*_*b*_, the fraction of the measured volume occupied by blood. rCPS is a function of the measured concentration of unlabeled leucine in arterial plasma, *C*_*p*_, and the rate constants*, i.e*.,

1 $$\mathrm{rCPS}\,=\,(\frac{{K}_{1}{k}_{4}}{{k}_{2}+\,{k}_{3}\,}){C}_{p}.$$

The fraction of unlabeled leucine in the precursor pool for protein synthesis derived from arterial plasma, $\lambda$, is given by

2 $$\lambda =\frac{{k}_{2}+\,{k}_{3}}{{k}_{2}+\,{k}_{3}+\,{k}_{4}}.$$

The remainder (1 − $\lambda$) derives from breakdown of unlabeled protein in tissue. In the present study the Basis Function Method (BFM) of Tomasi *et al*.^20^ was used for estimation of the kinetic model parameters of the homogeneous tissue model; the blood:brain equilibrium volume of distribution of ^11^CO_2_ (*V*_*D*_) is fixed at 0.41^21^. BFM utilizes a grid search approach together with linear least squares estimation. In the case that any kinetic model parameter estimate is negative for a particular voxel, estimates are replaced by those from the appropriately constrained linear least squares algorithm (Supplemental Material). The use of linear least squares algorithms results in faster and more robust estimation compared to the use of non-linear least squares methods. These properties are of high value when BFM is applied at voxel level, since it produces high quality parametric maps with reduced computational requirements^20^.

**Figure 2:**
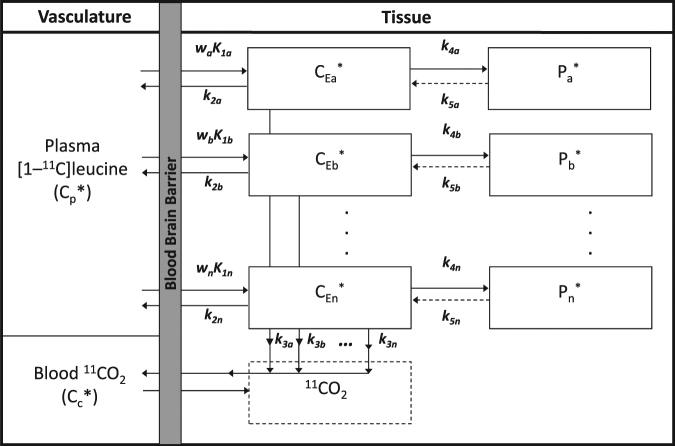
Heterogeneous Tissue Model for L-[1-^11^C]Leucine PET method. The heterogeneous tissue model^20^ for the labeled leucine assumed that each tissue is composed of *n* kinetically homogenous subregions (*a,b,…,n*), each described by the homogeneous tissue model of Fig. 1. The corresponding fractional weights of the subregions are *w*_*a*_ *w*_*b*_, … *w*_*n*_ (*w*_*a*_ ≥ 0, *w*_*b*_ ≥ 0, …, *w*_*n*_ ≥ 0; *n* ≥ *1; w*_*a*_ + *w*_*b*_ + … + *w*_*n*_ = 1). Total activity in the heterogeneous region as a whole can therefore be described by the weighted sum of the activities in each of the subregions. Expressed in terms of rate constants we have: $$\begin{array}{c}{C}_{T}^{\ast }(t)={\theta }_{0}{\int }_{0}^{t}{C}_{p}^{\ast }(\tau )d\tau +\,{\theta }_{a}{\int }_{0}^{t}{C}_{p}^{\ast }(\tau ){e}^{-{\beta }_{a}(t-\tau )}d\tau +\ldots +{\theta }_{n}{\int }_{0}^{t}{C}_{p}^{\ast }(\tau ){e}^{-{\beta }_{n}(t-\tau )}d\tau \\ \quad \quad \quad \quad +\,{V}_{b}[{C}_{b}^{\ast }(t)-{V}_{D}{C}_{c}^{\ast }(t)]+\,{V}_{D}{C}_{c}^{\ast }(t)\end{array}$$ where ${\theta }_{0}=(1-{V}_{b}){\sum }_{i=a}^{n}[\frac{{w}_{i}{K}_{1i}{k}_{4i}}{{k}_{2i}+{k}_{3i}+{k}_{4i}}],$ ${\beta }_{i}={k}_{2i}+{k}_{3i}+{k}_{4i},$and ${\theta }_{i}=(1-{V}_{b})[\frac{{w}_{i}{K}_{1i}({k}_{2i}+{k}_{3i})}{{k}_{2i}+{k}_{3i}+{k}_{4i}}]\,for\,i=a,\,b,\,\ldots ,\,n.$ If *n*, *θ*_0_, *θ*_*i*_ (*i* *=* *a*, *b*, *…*, *n*) and *V*_*b*_ are known, or have been estimated, then the above equations can be used to determine the weighted average influx rate constant for the mixed tissue, *K*_1_ = *w*_*a*_*K*_*1a*_ + *w*_*b*_*K*_*1*_ _*b*_+ *…* +*w*_*n*_*K*_*1n*_, as ${K}_{1}=\frac{{\theta }_{0}+\,{\sum }_{i=a}^{n}\,{\theta }_{i}}{(1-{V}_{b})}$. Note that we cannot identify the individual subregion’s weighted influx rate constant *w*_*i*_*K*_*1i*_, but only the sum over all subregions. Nor can the fraction of unlabeled leucine derived from arterial plasma in each individual subregion, i.e., *λ*_*i*_ = (*k*_*2i*_ + *k*_*3i*_)/(*k*_*2i*_ + *k*_*3i*_ + *k*_*4i*_), be identified as this fraction appears in the equation for *θ*_*i*_ only as the product with the non-identifiable term *w*_*i*_*K*_*1i*_. However, regional variations in measured values of *λ* are small^18^ and the parameter can be assumed constant across subregions, (i.e. *λ* = *λ*_a_ = *λ*_b_ = … = *λ*_*n*_), resulting in$\lambda =\frac{{\sum }_{i=a}^{n}{\theta }_{i}}{{\theta }_{0}\,+\,{\sum }_{i=a}^{n}{\theta }_{i}\,}$ Weighted average rCPS is then given by ${\rm{rCPS}}=(\frac{{w}_{a}{K}_{1a}{k}_{4a}}{{k}_{2a}\,+\,{k}_{3a}}+\frac{{w}_{b}{K}_{1b}{k}_{4b}}{{k}_{2b}\,+\,{k}_{3b}}+\ldots +\,\frac{{w}_{n}{K}_{1n}{k}_{4n}}{{k}_{2n}\,+\,{k}_{3n}}){C}_{p}={K}_{1}[\frac{1-\lambda }{\lambda }]{C}_{p}.$

The model for L-[1−^11^C]leucine that explicitly takes tissue heterogeneity into account^22^ is shown in Fig. 2. It assumes that each tissue is composed of *n* kinetically homogenous subregions whose weighted combination expresses total activity measured in the given volume. In the current study, estimation of parameters of interest, allowing that there may be more than one kinetically homogeneous subregion in the voxel, was performed with the Spectral Analysis Iterative Filter (SAIF) method^22,23^. SAIF was developed in order to quantify L-[1-^11^C]leucine PET data without prior assumptions concerning kinetic homogeneity or heterogeneity in the data^22^. SAIF is a modified version of Spectral Analysis used for [^18^F]fluorodeoxyglucose PET studies^24,25^. SAIF implements a filtering procedure to reduce the impact of noise; this makes voxel-wise quantification feasible^22^. The filter employed by SAIF is a bandpass filter that separates blood components and tracer trapped in the tissue from other tissue components. Additionally, the filter limits detection of phantom components that arise from measurement noise. For irreversible tracers SAIF is optimized for estimation of the weighted average rate constant for net trapping of tracer and the weighted average influx rate constant (*K*_1_), two parameters that are fundamental for the computation of $\lambda$ and rCPS^22,23^. In addition to the parameter estimates, SAIF returns the number of components detected in the tissue and thus provides information about the presence of kinetic inhomogeneity^22^. SAIF utilizes a non-negative linear least squares estimator, which is computationally faster than non-linear estimators, but slower than unconstrained linear least squares.

### Simulation Studies

Simulation studies were performed to evaluate the performance of BFM and SAIF under different kinetic modeling assumptions and different noise conditions. For simulating homogeneous tissue data, we began with the kinetic model parameters estimated in 18 regions of interest and whole brain in normal healthy volunteers (Bishu *et al*.^18^). The 19 inter-subject mean regional estimates of *K*_1_, *k*_2_ + *k*_3_ and *k*_4_ were used as our reference parameter set; *V*_*b*_ was fixed at 0.05. Values of *K*_1_ in the reference set were between 0.025 and 0.062 mL/g/min, *k*_2_ + *k*_3_ ranged from 0.054 to 0.157 min^−1^, and *k*_4_ from 0.027 to 0.053 min^−1^. For each set of reference parameters we computed the corresponding values of rCPS and $\lambda$ by use of equations (1) and (2), respectively, and generated a tissue time-activity curve based the equation of the homogeneous tissue model (Fig. 1). This case was our *homogenous tissue simulation scenario*.

From each set of parameters in the homogeneous tissue reference set, we generated a reference set of kinetic model parameters for a heterogeneous tissue as follows. We assumed the heterogeneous tissue region had two homogeneous subregions of equal weight, and that values of *λ* were equal in the two subregions. *V*_*b*_ was kept at 0.05. We also assumed that the weighted average of the *K*_1_ values in the two subregions equals the *K*_1_ for the corresponding region in the homogeneous tissue simulation scenario. These assumptions assure that the weighted average rCPS in the mixed tissue equals rCPS in the corresponding homogeneous tissue. The remaining parameters were derived assuming that the sum of the rate constants *k*_2_ + *k*_3_ + *k*_4_ in the first subregion is five times that in the second subregion, and that the product of rate constants *K*_1_(*k*_2_ + *k*_3_) in the first subregion is 25 times that in the second subregion. These combinations of rate constants appear in the equation for total activity described by the heterogeneous tissue model (Fig. 2) and the values used in the simulation are consistent with previous analyses^22^. This case was our *heterogeneous tissue simulation scenario*.

All simulations used the arterial input functions from one subject studied previously^18^. For simplicity, ^11^CO_2_ in brain was not included in the simulations as it constitutes only 1–2% of the total measured activity^18^. The two sets of 19 previously generated time-activity curves were sampled as 42 frames of data (16 × 15, 4 × 30, 4 × 60, 4 × 150, 14 × 300 sec), the standard protocol in L-[1-^11^C]leucine PET studies^18,19^. For each frame of each reference time-activity curve (both homogenous and heterogeneous scenarios) 200 noise-added realizations were generated via Monte Carlo techniques. Noise was defined consistent with a stochastic process with zero mean and Gaussian distribution whose variance is described by

3 $$Var\,[{C}_{T}^{\ast }({t}_{i})]=\alpha {s}_{i}^{2}=\alpha \frac{\exp (\gamma {t}_{i})\,{C}_{T}^{\ast }({t}_{i})}{{\rm{\Delta }}{t}_{i}}$$

where $\gamma$ is the decay constant for ^11^C, *Δt*_*i*_ is the length of Frame *i*, *C*_T_^*^(t_*i*_) is the mean decay-corrected concentration of ^11^C in Frame *i* of the tissue, and $\alpha$ is the proportionality coefficient reflecting the noise level in the data^26^. The scale factor for the noise variance was set to be consistent with voxel-wise error, as detailed previously^20^, for both homogeneous and heterogeneous tissue simulation scenarios. Additionally, we generated noise consistent with regional time-activity curve error^22^ for the heterogeneous tissue simulation scenario. ROI-level noise was not used in the homogeneous tissue simulation scenario, assuming that a volume sufficient to have the lower noise level would be too large to contain only kinetically homogeneous tissue.

BFM and SAIF were then applied to all simulated datasets. For both methods a predefined grid of 100 values of *β* = *k*_2_ + *k*_3 _+ *k*_4_, logarithmically distributed^27^ in the range 0.0037–1.33 min^−1^, was used. For SAIF, the filter bandpass was set to [0.02 min^−1^, 0.3 min^−1^] in order to be consistent with application to low as well as high signal-to-noise ratios^23^. Weights were set proportional to the inverse of the error variance (equation (3)).

The performances of BFM and SAIF were evaluated in terms of robustness and accuracy. Robustness was defined as the number of failures to converge to a solution, or to find physiologically meaningful parameter estimates, divided by the total number of simulated cases^23^. We defined as non-physiological those estimates in which *K*_1_ > 1 mL g^−1^ min^−1^, *V*_b_ > 1, or *rCPS* > 10 nmol g^−1^ min^−1^. (The BFM and SAIF algorithms guarantee that all estimates are non-negative). For each of the 19 sets of parameters in the reference data set, accuracy was assessed as the bias in *K*_1_, $\lambda$ and rCPS, defined as the mean relative difference between simulated and estimated parameter values. Overall method performance was assessed as the root mean square error

4 $$RMS{E}_{j}=\sqrt[2]{\frac{1}{200}{\sum }_{i=1}^{200}{({p}_{j}-{\hat{p}}_{ij})}^{2}}$$

where ${p}_{j}$ represents the *j*^*th*^ simulated reference value for *K*_1_, $\lambda$ or rCPS (*j*=*1, 2, …*, *19*) and ${\hat{p}}_{{ij}}$ represents its *i*^*th*^ corresponding simulation estimate. For SAIF we also investigated the capacity of the method to correctly identify the number of subregions in the simulated tissue.

### Studies of L-[1-^11^C]leucine data measured with the High Resolution Research Tomograph

Data from previously-reported studies^18,19^ of eight healthy, awake, male subjects (age 20–24) were reanalyzed in the current study. The subjects were chosen to have a range of injected activities of L-[1-^11^C]leucine. Five of our reanalyzed group were administered >0.30 mCi L-[1-^11^C]leucine/kg body weight, the target dose for the studies. The remaining 3 subjects had injected doses of 0.29, 0.17, and 0.16 mCi/kg. Subject inclusion criteria as well as the PET scan protocol were described in detail previously^18^. Briefly, all PET studies were performed on the ECAT High Resolution Research Tomograph (HRRT) (CPS Innovations, Knoxville, TN), with a spatial resolution of ~2.6 mm full width at half maximum (FWHM)^28^. The 90-min emission scan was initiated coincident with a 2-min intravenous infusion of L-[1-^11^C]leucine. Images were reconstructed using the motion-compensated 3D ordinary Poisson ordered subset expectation maximization algorithm^28^ as 42 frames of data (16 × 15, 4 × 30, 4 × 60, 4 × 150, 14 × 300 sec); voxel size was 1.21 × 1.21 × 1.23 mm. Arterial blood was sampled over the course of the study and concentrations of unlabeled and labeled leucine in arterial plasma and total ^11^C and ^11^CO_2_ activities in whole blood were measured according to methods detailed previously^18^. All subjects underwent a T1-weighted MRI of the brain. ROIs were drawn on the MRI, and a 3D volume of each ROI mask was created. The MRI was then co-registered to the mean of the 30–60 min PET images by use of a 3D rigid body transformation and the transformation parameters were applied to the ROI mask volumes. Vinci software (Volume Imaging in Neurological research, Co-registration and ROIs Included; Max Planck Institute for Neurological Research, Cologne, Germany) was used for the co-registration.

Whole brain and 12 regions (specifically cerebellum, caudate, putamen, frontal cortex, corona radiata, thalamus, occipital cortex, parietal cortex, vermis, amygdala, hippocampus and temporal cortex) were evaluated in all subjects. Estimates of *K*_1_, *λ*, and rCPS were determined in each voxel by means of both BFM and SAIF. The algorithm settings were maintained the same as in the simulation studies. The difference between tracer arrival time in brain and arterial sampling site was estimated by shifting the whole brain time-activity curve (TAC) with various tracer delays (0–20 sec), fitting the whole brain TAC by use of SAIF, and selecting the delay that produced the smallest weighted residual sum of squares.

For all subjects and regions, the mean of the voxel estimates in all voxels of the ROI was used as the parameter estimate for the region. Correlation between SAIF and BFM estimates was assessed, and the relative difference between BFM and SAIF estimates was computed. In addition, we used SAIF to assess the level of heterogeneity for each region, defined as the fraction of voxels described by two or more equilibrating components. Analysis was performed by single application of SAIF and by use of a bootstrap approach (N of repetitions = 50)^23^. The method exploits random resampling of normalized residuals as an alternative to random noise generation with a Gaussian model^29,30^. Heterogeneity maps were constructed by fusing the MR image, resliced to match the PET data, with a map of the spatial distribution of voxels with two or more SAIF-estimated equilibrating components.

### Influence of PET scanner resolution

In order to examine the influence of scanner resolution on the L-[1-^11^C]leucine data analyses, we artificially reduced the resolution of our data by applying a 3D Gaussian filter (FWHM 7.1 mm) to all HRRT PET images in the study. The decreased-resolution data were reanalyzed with both SAIF and BFM, and the same comparisons were made as in the unsmoothed HRRT data described above.

### Availability of Datasets

Datasets generated during and/or analyzed in the current study are available from the corresponding author upon request.

## Results

### Simulated data: Comparison of BFM and SAIF performance

BFM and SAIF demonstrated a high degree of robustness to noise in terms of producing physiologically meaningful parameter estimates. We define outliers as cases in which the algorithm fails to converge to physiologically meaningful estimates. Less than 1% of the total simulated TACs produced outliers when analyzed by SAIF, independent of the simulation scenario and level of noise (Outlier Fraction, Table 1). At high noise levels BFM returned ~1% outliers for both homogenous and heterogeneous model scenarios. At low noise levels, less than 1% of the BFM estimates were outliers. These results are in agreement with previous findings^20,23^.

**Table 1:** Performance of BFM and SAIF algorithms in simulation studies^a^.

Simulation Scenario	Analysis	*K*_1_				*λ*				rCPS				Outliers^b^	
		Bias^c^ (%)		RMSE^d^ (%)		Bias^c^ (%)		RMSE^d^ (%)		Bias^c^ (%)		RMSE^d^ (%)		(% of total)	
		Noise Level^e^		Noise Level^e^		Noise Level^e^		Noise Level^e^		Noise Level^e^		Noise Level^e^		Noise Level^e^	
		ROI	Voxel	ROI	Voxel	ROI	Voxel	ROI	Voxel	ROI	Voxel	ROI	Voxel	ROI	Voxel
Homogeneous Tissue	BFM		1 ± 1		8 ± 1		<1 ± 2		6 ± 2		1 ± 4		20 ± 5		<1 ± <1
	SAIF		2 ± 1		8 ± 1		2 ± 1		8 ± 1		−4 ± 4		23 ± 4		<1 ± < 1
Heterogeneous Tissue	BFM	1 ± 1	<1 ± 1	2 ± <1	8 ± 1	−5 ± 1	−3 ± 2	5 ± 1	7 ± 2	15 ± 5	13 ± 5	16 ± 4	26 ± 5	< 1 ± < 1	1 ± 2
	SAIF	1 ± 1	2 ± 1	2 ± 1	8 ± 1	−1 ± 1	<1 ± 2	3 ± 1	8 ± 2	5 ± 4	5 ± 5	10 ± 2	26 ± 5	<1 ± <1	<1 ± < 1

^a^Values are Means ± SD for the 19 sets of reference parameters.

^b^Outliers are those cases in which the algorithm did not converge to a solution or led to nonphysiological estimates.

^c^Bias was computed as the mean relative difference between the true simulated and estimated parameter values.

^d^RMSE was computed as the square root of the mean of the square of all of the error.

^e^Noise levels are consistent with Region-of-Interest (ROI) or voxel-level data.

**Figure 3:**
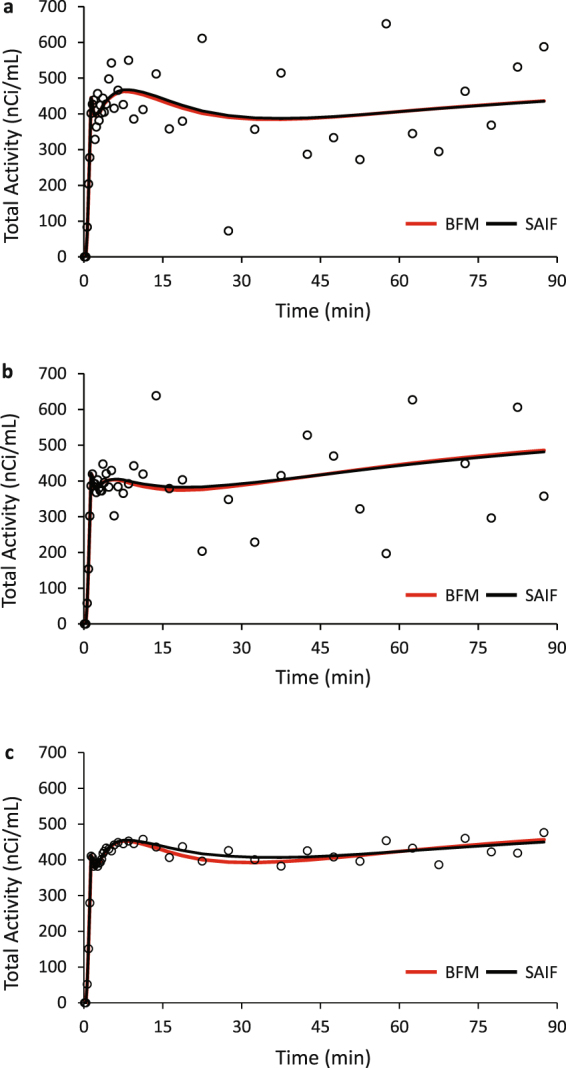
Simulated Time-Activity Curves. (**a**) Simulated homogeneous tissue TAC with noise variance consistent with voxel error. (**b**) Simulated heterogeneous tissue TAC with noise variance consistent with voxel error. (**c**) Simulated heterogeneous tissue TAC with noise variance consistent with ROI error. Open circles denote simulated data points, red line is the best-fitting TAC from BFM estimation, and black line is the best-fitting TAC from SAIF estimation.

Percentage bias and RMSE for the parameters *K*_1_, *λ*, and rCPS are shown in Table 1. Values represent the mean ± standard deviation for the 19 sets of simulated parameters. In general, performance of the estimation methods reflected the relationship between the type of simulated kinetics and the kinetic model assumptions on which BFM and SAIF are based. BFM performed well in the homogenous tissue simulation scenario with noise consistent with voxel level data (see TAC, Fig. 3a). When the method was applied to the heterogeneous tissue simulation scenario with voxel-level noise (see TAC, Fig. 3b), bias and RMSE in *K*_*1*_ and *λ* were similar to the homogeneous tissue simulation, but bias and RMSE in rCPS were higher. With low ROI-level noise in the heterogeneous tissue simulation (see TAC, Fig. 3c), overall performance of BFM was worse in terms of bias but better in terms of RMSE. This behavior (higher bias for lower noise) suggests that the misspecified tracer kinetic model (homogeneous tissue model applied to a heterogeneous tissue) is mainly responsible for the poor performance.

SAIF, on the other hand, showed very similar bias and RMSE in the homogeneous tissue and heterogeneous tissue simulation scenarios with high voxel-level noise. In the heterogeneous tissue simulation scenario with high voxel-level noise, SAIF biases in all the parameters were lower than the corresponding BFM biases, but *RMSE in all parameters were equivalent for BFM and SAIF*. In the heterogeneous tissue simulation scenario with low ROI-level noise, bias in SAIF estimates was ~1% for *K*_*1*_ and *λ*, and 5% for rCPS.

The capacity of SAIF for measuring the degree of heterogeneity was influenced by the level of noise. At the low regional noise level SAIF returned the correct degree of heterogeneity in 89% of simulated cases. Mis-classified cases were almost completely associated with SAIF’s finding a higher degree of heterogeneity than the simulated one (i.e., number of subregions >2). This tendency of SAIF to overestimate the number of tissue components was amplified at the high noise level: only 49% of the homogeneous simulated spectra were correctly identified, while 49% and 2% were associated with 2 and 3 equilibrating components, respectively. Of the heterogeneous simulated spectra, 79% were correctly identified, while the remaining ~20% were associated with 3 or more equilibrating components. Less than 1% of voxels with simulated heterogeneous kinetics were identified by SAIF as homogeneous. These results are in agreement with what was observed with standard spectral analysis technique^31^ and previous SAIF analyses^23^.

### High resolution measured data: Comparison of BFM and SAIF performance

**Figure 4:**
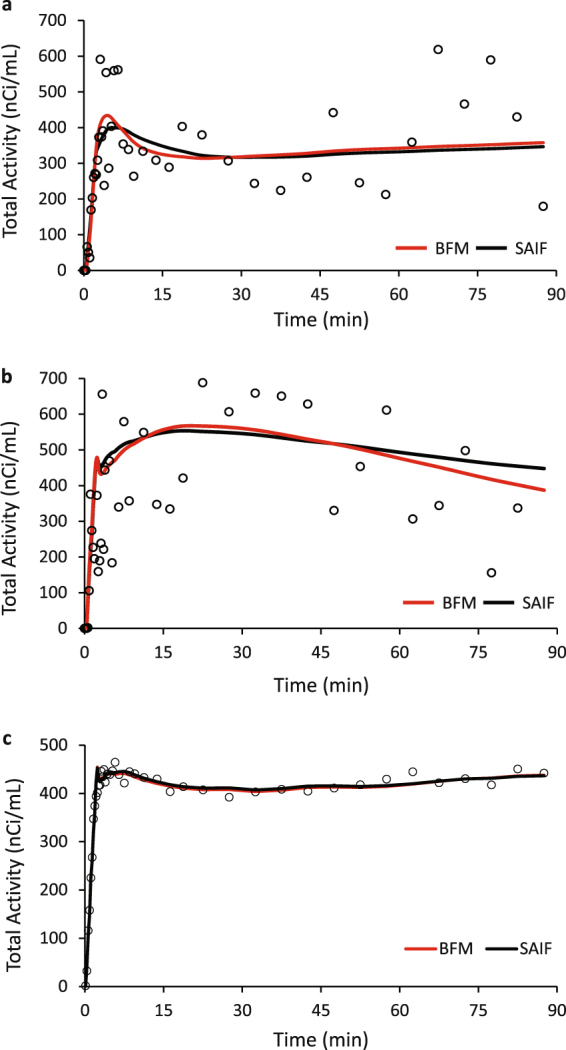
Time-Activity curves measured with the High Resolution Research Tomograph. (**a**) Single voxel TAC in which BFM and SAIF methods yielded good agreement in rCPS (relative difference < 1%). (**b**) Single voxel TAC in which BFM and SAIF methods yielded different estimates for rCPS. BFM returned a zero value for rCPS and SAIF a positive one. Open circles are measured data points, red line is the best-fitting TAC from BFM estimation, and black line is the best-fitting TAC from SAIF estimation.(**c**) Representative time-activity curve of a 15 cc region drawn on the parietal cortex of one subject. Open circles are the mean of ~8300 measured voxel TACs. Means of the best fitting BFM and SAIF curves of the individual voxel TACs are shown as red and black lines, respectively. After the first minute, the difference between the two best-fitting curves was <1%. In this region, the methods also showed good agreement in the mean of the voxel estimates of rCPS (<4%).

**Figure 5:**
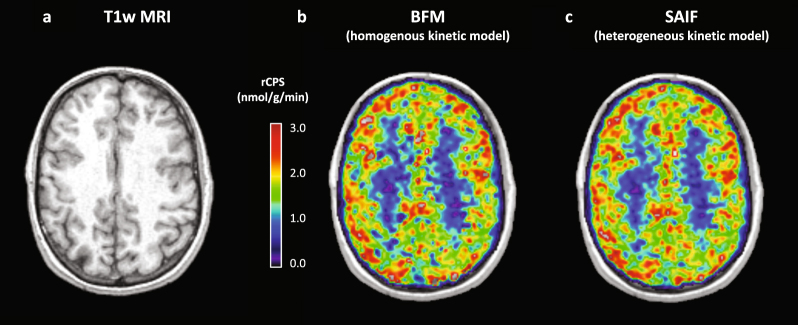
rCPS estimated with BFM and SAIF. (**a**) MRI T1-weighted image. (**b**) rCPS parametric map obtained with BFM (under the hypothesis of a homogenous tissue kinetic model). (**c**) rCPS estimated with SAIF (under the hypothesis of a heterogeneous tissue kinetic model). Data are from a single 21 year old male subject who underwent a L-[1-^11^C]leucine PET study with the High Resolution Research Tomograph (injected dose 0.39 mCi/kg).

**Figure 6:**
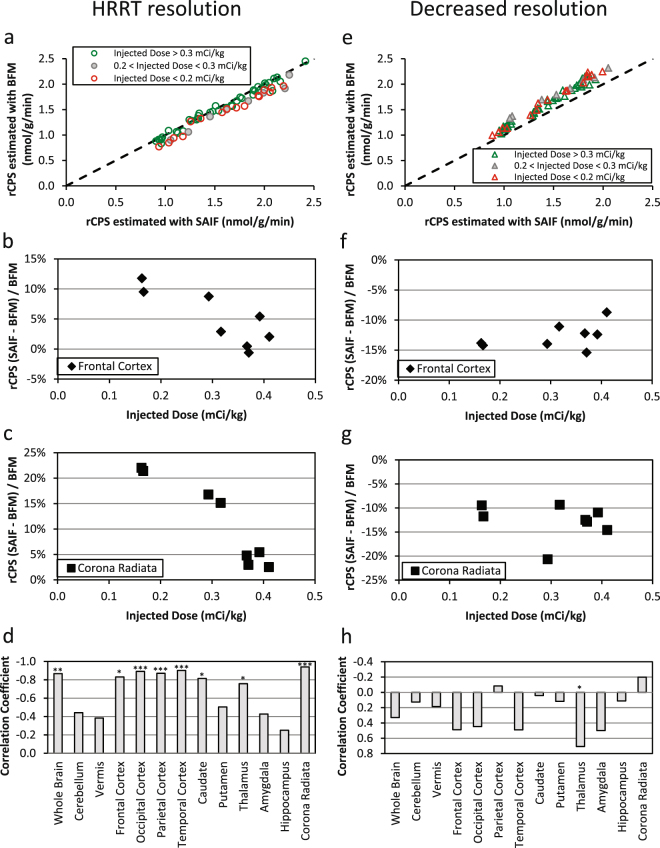
Effect of injected dose of L-[1-^11^C]leucine on differences in rCPS estimated with BFM and SAIF. (**a**) Regional values of rCPS estimated with SAIF (abscissa) and BFM (ordinate) from a L-[1-^11^C]leucine PET study with the High Resolution Research Tomograph (HRRT). Each circle represents one region in one subject; results from eight subjects; whole brain and 7 regions are shown (cerebellum, frontal and occipital cortices, thalamus, caudate, putamen, and corona radiata). Green circles are regions in five subjects with injected doses >0.3 mCi/kg L-[1-^11^C]leucine, red circles are regions in two subjects with injected dose <0.2 mCi/kg, and gray-filled circles are regions in one subject with an intermediate dose. Dotted line is the line of identity. (**b**,**c**) Relative difference between rCPS estimated with the BFM and SAIF methods in the frontal cortex and corona radiata, respectively. Differences (ordinate) are shown for the various doses (abscissa) injected in our subjects. Studies were carried out on the HRRT which has a spatial resolution of ~2.6 mm FWHM^40^. Note the tendency for lower injected doses to be associated with higher differences in rCPS between estimation methods. (**d**) Correlations between injected dose and relative difference in rCPS between methods for 12 regions and whole brain (*p < 0.05; **p < 0.01; ***p < 0.005). Correlations are negative reflecting increases in relative differences in rCPS with decreases in injected dose. (**e**–**h**) Results corresponding to b-d from analysis of HRRT emission data spatially smoothed to simulate scans from a lower spatial resolution tomograph. With reduced spatial resolution BFM estimates of rCPS are systematically higher in all regions and subjects than rCPS estimated with SAIF, and the correlation between relative differences in rCPS and injected dose is not significant in most regions.

**Figure 7:**
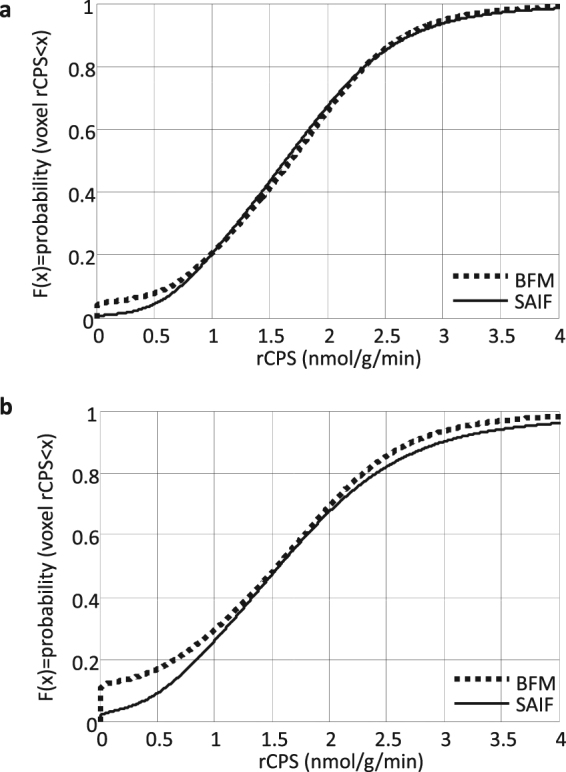
Cumulative distribution of voxel values of rCPS estimated with BFM and SAIF. On the abscissa are values of rCPS in the range 0–4 nmol/g/min; estimated rCPS in most voxels in whole brain fall into this interval. On the ordinate is the fraction of all voxels in brain in which estimated rCPS is less than or equal to the corresponding value on the abscissa. (**a**) data from the subject with the highest injected dose (0.41 mCi/kg L-[1-^11^C]leucine) and (**b**) subject with the lowest injected dose (0.16 mCi/kg). In the high dose subject, the largest differences in rCPS estimated with the two methods are in the voxels with rCPS < 1 nmol/g/min; these are voxels concentrated in white matter. In the low dose subject, differences in rCPS estimated with the two methods are greater at both ends of the range of rCPS values. Estimates of rCPS were made from the data acquired on the HRRT scanner. Number of voxels in brain, from which the empirical cumulative distribution functions were determined, is approximately 0.7 × 10^6^.

Both BFM and SAIF were highly robust; the methods converged to physiological solutions in more than 99% of the voxels analyzed. In terms of fit of the data (Fig. 4), SAIF provided a slightly better description of the voxel TACs compared with BFM, as indicated by lower values of the weighted residual sums of squares (mean relative difference: −7%). This result was expected since SAIF is based on an over-complete representation of the system that has a higher number of degrees of freedom than BFM^32^. Qualitatively, parameters estimated with SAIF and BFM applied to HRRT L-[1-^11^C]leucine PET data showed good agreement; rCPS is shown in Fig. 5. Quantitatively, the means of parameter estimates taken over all voxels in each ROI showed good agreement between methods in the parameter *K*_1_ (not shown) and in rCPS (Fig. 6a), but *λ* was slightly higher when estimated with BFM (not shown). Mean regional estimates of rCPS computed by BFM and SAIF, however, showed a tendency to agree better in those subjects who were administered larger doses of L-[1-^11^C]leucine (≥0.3 mCi/kg, green circles, Fig. 6a) than in those administered lower doses (<0.3 mCi/kg, red and gray circles, Fig. 6a). This was due to slightly lower mean BFM-estimated rCPS, and slightly higher mean SAIF-estimated rCPS, in the subjects with lower doses compared with the subjects who had higher injected doses; none of the differences reached statistical significance, with the exception of rCPS estimated with SAIF in thalamus (p < 0.05, Student’s *t*-test). We produced scatter plots to further examine the relationship between injected dose of L-[1-^11^C]leucine and the percentage difference between rCPS determined by the two methods (Fig. 6b,c). In many regions there was a clear trend for better agreement between rCPS estimates at higher injected doses. Furthermore, the absolute difference in rCPS estimates became quite small at high injected doses. In whole brain, cortex, caudate, thalamus and white matter there was a statistically significant negative linear correlation between injected dose of L-[1-^11^C]leucine and the difference between rCPS estimated by the two methods (Fig. 6d). To see which voxels produce the largest difference in rCPS estimated by the two methods, we look at their respective cumulative distribution functions (CDFs, Fig. 7). Figure 7a illustrates the CDFs for BFM-estimated and SAIF-estimated rCPS in all brain voxels for the subject with the highest injected dose of L-[1-^11^C]leucine. The largest difference between rCPS estimates occurs when rCPS is low (<1 nmol/g/min); this implies we would expect higher differences in rCPS between methods in regions that are high in white matter content, since white matter has low rCPS. In the subject with the lowest injected dose (Fig. 7b) differences in rCPS between methods are greater at low values of rCPS. Additionally, the entire BFM CDF lies to the left of that of SAIF, indicating overall lower estimates of rCPS with BFM.

The foregoing data show that differences in rCPS estimated with BFM and SAIF are dissimilar in the subjects who received a low injected dose from those who received higher doses. We, therefore, looked at how the methods compare in the subset of five subjects who received a dose of L-[1-^11^C]leucine greater than or equal to the target dose for our studies of 0.30 mCi/kg (Table 2). Estimates of *K*_1_, *λ*, and rCPS for each region and each subject were determined as the mean of the parameter estimates taken over all voxels within the region. Data in Table 2 are the inter-subject means and SDs of the regional parameter estimates for the five subjects. Estimates of *λ* demonstrated the closest agreement between methods, with relative differences ranging from −6% to +1%, depending on the region. *K*_1_ also showed good agreement (relative difference between methods −10% to +2%). In general rCPS estimated with SAIF was higher than when estimated with BFM. In amygdala and hippocampus the discrepancy between methods was greatest: rCPS estimated with SAIF was 13% and 15% higher, respectively, than when estimated with BFM. In cerebellum and corona radiata SAIF estimates were 6% higher, and in all other regions the difference was <5%. There was good agreement between SAIF and BFM estimates in terms of variability.

**Table 2:** Parameters estimated from data measured with the High Resolution Research Tomograph^a^.

	*Volume* (cc)	Spectral Analysis Iterative Filter (SAIF)			Basis Function Method (BFM)			
		*K*_1_ (ml/g/min)	*λ* (unitless)	*rCPS* (nmol/g/min)	*K*_1_ (ml/g/min)	*λ* (unitless)	*rCPS* (nmol/g/min)	(SAIF-BFM)/BFM^b^ rCPS
HRRT Resolution								
*Whole Brain*	1411.2 ± 179.3	0.050 ± 0.008	0.75 ± 0.03	1.69 ± 0.05	0.052 ± 0.008	0.77 ± 0.02	1.62 ± 0.08	4.3% ± 2.4%
*Cerebellum*	118.2 ± 12.0	0.063 ± 0.011	0.75 ± 0.02	2.04 ± 0.10	0.070 ± 0.011	0.78 ± 0.02	1.93 ± 0.14	6.0% ± 4.8%
*Vermis*	5.4 ± 1.1	0.060 ± 0.012	0.74 ± 0.03	2.01 ± 0.15	0.065 ± 0.013	0.77 ± 0.03	1.91 ± 0.14	4.8% ± 5.2%
*Cortical Regions*								
*Frontal Cortex*	122.8 ± 24.1	0.053 ± 0.009	0.74 ± 0.03	1.88 ± 0.06	0.054 ± 0.009	0.75 ± 0.02	1.84 ± 0.09	2.0% ± 2.3%
*Occipital Cortex*	30.9 ± 15.3	0.063 ± 0.010	0.74 ± 0.02	2.12 ± 0.17	0.066 ± 0.010	0.75 ± 0.03	2.12 ± 0.19	0.0% ± 1.2%
*Parietal Cortex*	14.2 ± 4.5	0.054 ± 0.009	0.74 ± 0.02	1.92 ± 0.12	0.056 ± 0.008	0.76 ± 0.02	1.87 ± 0.14	2.9% ± 2.0%
*Temporal Cortex*	54.4 ± 10.7	0.049 ± 0.008	0.73 ± 0.02	1.79 ± 0.06	0.049 ± 0.008	0.75 ± 0.02	1.71 ± 0.07	4.2% ± 2.4%
*Subcortical Gray*								
*Caudate*	7.9 ± 1.2	0.039 ± 0.008	0.78 ± 0.03	1.08 ± 0.08	0.038 ± 0.007	0.79 ± 0.02	1.04 ± 0.05	3.8% ± 5.9%
*Putamen*	8.9 ± 1.2	0.053 ± 0.010	0.80 ± 0.01	1.30 ± 0.05	0.052 ± 0.009	0.79 ± 0.02	1.34 ± 0.06	−2.8% ± 2.6%
*Thalamus*	18.9 ± 3.2	0.049 ± 0.009	0.77 ± 0.02	1.46 ± 0.09	0.051 ± 0.009	0.78 ± 0.02	1.45 ± 0.10	0.5% ± 2.9%
*Amygdala*	4.9 ± 0.7	0.035 ± 0.004	0.71 ± 0.03	1.49 ± 0.06	0.036 ± 0.004	0.75 ± 0.02	1.32 ± 0.08	13.4% ± 7.3%
*Hippocampus*	4.9 ± 1.7	0.038 ± 0.006	0.72 ± 0.03	1.54 ± 0.07	0.039 ± 0.006	0.76 ± 0.02	1.35 ± 0.14	14.5% ± 7.0%
*White Matter*								
*Corona Radiata*	47.6 ± 6.9	0.032 ± 0.006	0.77 ± 0.03	0.94 ± 0.02	0.031 ± 0.006	0.79 ± 0.02	0.89 ± 0.03	6.2% ± 5.2%

^a^All subjects with injected dose ≥ 0.30 mCi/kg body weight; values are inter-subject mean ± SD, n = 5.

^b^Values are inter-subject mean ± SD of the relative difference in each subject.

It is interesting to note that the methods require substantially different computational power: SAIF required at least 4.5 hours for a whole brain analysis while BFM averaged about 30 minutes. Times refer to analyses run on the same desktop computer (Intel® Core™ i7-3770 CPU @ 3.40 GHz, RAM 32 GB), without parallel computing optimization.

### Decreased resolution of measured data: Comparison of BFM and SAIF performance

PET data resolution had a strong impact on estimates of the parameters of interest for both methods of estimation (Table 3). When SAIF was used in the analysis, reduced spatial resolution led to decreased mean regional estimates of *K*_1_ by 4–10% (except in corona radiata) and decreased estimates of rCPS by 2–16% (except in corona radiata and putamen); estimates of *λ* remained largely unchanged. In all regions other than corona radiata, analysis with BFM also led to lower estimates of *K*_1_ by 7–19%, lower estimates of *λ* by 3–10%, and higher estimates of rCPS by 4–15% than found with the original HRRT resolution data.

**Table 3:** Parameters estimated from measured PET data smoothed to lower spatial resolution^a^.

	*Volume* (cc)	Spectral Analysis Iterative Filter (SAIF)			Basis Function Method (BFM)			
		*K*_1_ (ml/g/min)	*λ* (unitless)	*rCPS* (nmol/g/min)	*K*_1_ (ml/g/min)	*λ* (unitless)	*rCPS* (nmol/g/min)	(SAIF-BFM)/BFM^b^ rCPS
Reduced Resolution (Smoothed Gaussian Kernel 7 mm FWHM)								
*Whole Brain*	1411.2 ± 179.3	0.046 ± 0.008	0.75 ± 0.03	1.56 ± 0.04	0.045 ± 0.009	0.72 ± 0.04	1.77 ± 0.06	−12.0% ± 2.5%
*Cerebellum*	118.2 ± 12.0	0.059 ± 0.011	0.77 ± 0.02	1.78 ± 0.07	0.057 ± 0.010	0.74 ± 0.03	2.01 ± 0.10	−11.1% ± 3.3%
*Vermis*	5.4 ± 1.1	0.055 ± 0.011	0.76 ± 0.03	1.68 ± 0.10	0.053 ± 0.011	0.73 ± 0.03	1.90 ± 0.15	−11.5% ± 4.3%
*Cortical Regions*								
*Frontal Cortex*	122.8 ± 24.1	0.049 ± 0.009	0.74 ± 0.03	1.76 ± 0.03	0.048 ± 0.010	0.70 ± 0.04	1.99 ± 0.07	−12.0% ± 2.4%
*Occipital Cortex*	30.9 ± 15.3	0.059 ± 0.010	0.76 ± 0.03	1.91 ± 0.10	0.056 ± 0.010	0.72 ± 0.04	2.22 ± 0.17	−13.6% ± 3.4%
*Parietal Cortex*	14.2 ± 4.5	0.049 ± 0.008	0.74 ± 0.02	1.71 ± 0.07	0.047 ± 0.008	0.70 ± 0.04	1.97 ± 0.13	−13.1% ± 3.6%
*Temporal Cortex*	54.4 ± 10.7	0.044 ± 0.008	0.73 ± 0.03	1.65 ± 0.05	0.043 ± 0.009	0.70 ± 0.04	1.86 ± 0.06	−11.3% ± 2.4%
*Subcortical Gray*								
*Caudate*	7.9 ± 1.2	0.035 ± 0.007	0.77 ± 0.03	1.03 ± 0.05	0.035 ± 0.009	0.74 ± 0.04	1.17 ± 0.09	−12.4% ± 4.1%
*Putamen*	8.9 ± 1.2	0.049 ± 0.009	0.79 ± 0.02	1.32 ± 0.03	0.049 ± 0.010	0.77 ± 0.03	1.47 ± 0.06	−10.2% ± 2.3%
*Thalamus*	18.9 ± 3.2	0.047 ± 0.010	0.77 ± 0.02	1.42 ± 0.07	0.045 ± 0.009	0.74 ± 0.03	1.62 ± 0.10	−12.0% ± 2.7%
*Amygdala*	4.9 ± 0.7	0.032 ± 0.005	0.70 ± 0.03	1.37 ± 0.07	0.032 ± 0.007	0.68 ± 0.05	1.50 ± 0.07	−9.1% ± 1.6%
*Hippocampus*	4.9 ± 1.7	0.035 ± 0.007	0.72 ± 0.03	1.39 ± 0.10	0.034 ± 0.009	0.69 ± 0.05	1.55 ± 0.07	−10.4% ± 4.5%
*White Matter*								
*Corona Radiata*	47.6 ± 6.9	0.032 ± 0.007	0.76 ± 0.03	1.01 ± 0.04	0.031 ± 0.008	0.73 ± 0.05	1.14 ± 0.05	−12.0% ± 2.0%

^a^All subjects with injected dose ≥0.30 mCi/kg body weight; values are inter-subject mean ± SD, n = 5.

^b^Values are inter-subject mean ± SD of the relative difference in each subject.

Application of spatial filtering to reduce the PET resolution changed the sign of the differences between SAIF and BFM parameter estimates, most notably rCPS. This can be seen in the scatter analysis (Fig. 6e) as well as in Table 3. Estimates of *K*_1_ were 1% to 6% higher, estimates of *λ* were 3% to 5% higher, and estimates of rCPS were 9% to 14% lower when estimated with SAIF than when estimated with BFM. There was no apparent relationship between injected dose of L-[1-^11^C]leucine and differences in rCPS estimated with the two methods (Fig. 6f,g,h).

### Analysis of kinetic heterogeneity

**Figure 8:**
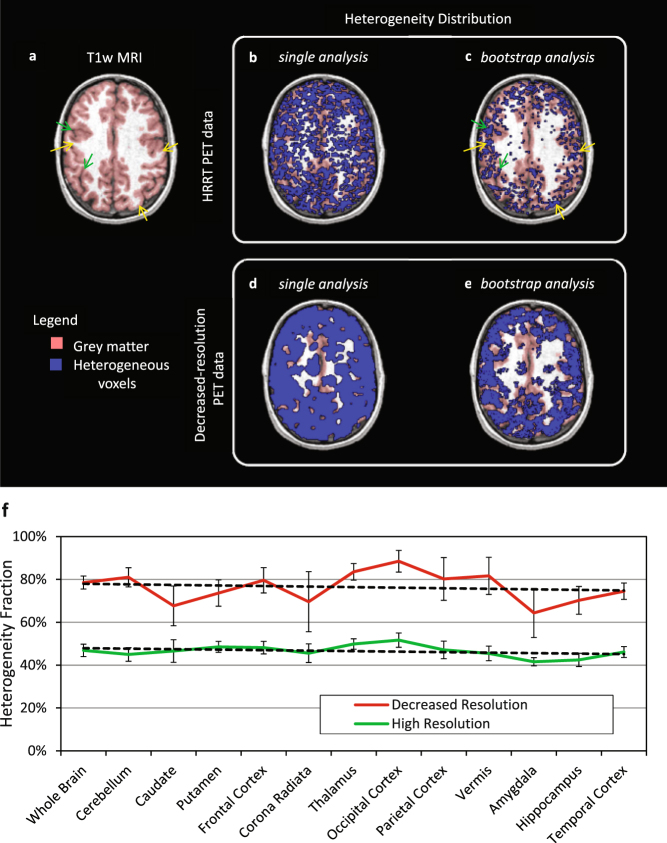
Distribution of kinetic inhomogeneity estimated by SAIF. T1-weighted MRI image (**a**) and the same image fused with an image of the spatial distribution of voxels estimated to be heterogeneous (Panels b–e). Panels b and c refer to HRRT PET data with single and bootstrap application of SAIF, respectively. Panels d and e refer to the corresponding single and bootstrap applications of SAIF with decreased-resolution PET data. The blue areas represent voxels where the SAIF algorithm detects at least two equilibrating components in the voxel time-activity curve. The red area highlights gray matter, including voxels at its border with white matter. For the bootstrap approach the probability threshold for heterogeneity voxel selection was fixed to 0.80 (see text). In the HRRT resolution PET data (**c**), the heterogeneous voxels appear to be concentrated at the borders between gray and white matter (see, for example, yellow arrows) as well as in some sulci (see, for example green arrows). The areas with heterogeneous voxels expand considerably when the resolution of the image is decreased (**e**). Panel f illustrates the effect of resolution on detection of kinetic inhomogeneity. Green and red lines represent, respectively, the mean heterogeneity fraction determined in an SAIF single analysis of each ROI in the HRRT and decreased-resolution PET data. Error bars are the standard deviation of the regional heterogeneity fractions in the eight subjects.

The heterogeneity fraction (HF) for each region was computed by SAIF as the number of voxels found to have two or more subregions divided by the total number of voxels in the region. Maps of the heterogeneity fraction for one subject are shown in Fig. 8. This fraction showed an inverse relationship between image resolution and degree of heterogeneity: in the original HRRT PET data the whole brain heterogeneity fraction was 47% ± 2% (inter-subject mean ± SD, n = 8); in the decreased-resolution PET data the corresponding fraction increased to 76% ± 6% (inter-subject mean ± SD, n = 8). There were no apparent differences in the heterogeneity fraction among the regions analyzed (Fig. 8f) or between the subjects injected lower doses of L-[1-^11^C]leucine (data not shown). From a single analysis of each subject, it was not possible to localize which areas in brain have higher heterogeneity fractions (Fig. 8b,d). When the heterogeneity fraction was assessed with a bootstrap approach^23^, however, a clearer distribution pattern became apparent (Fig. 8c,e). In the HRRT resolution PET data, the heterogeneous voxels appear to be concentrated at the borders between gray and white matter and in the sulci (Fig. 8c); the border areas of heterogeneous voxels expand considerably when the resolution of the image is decreased (Fig. 8e).

## Discussion

In this work we explored the impact of tissue kinetic heterogeneity on PET parametric mapping of *in vivo* cerebral protein synthesis rates, with the final aim of understanding whether this effect is trivial or is instead a real source of bias for voxel-by-voxel analyses. We used L-[1-^11^C]leucine PET data acquired on a high resolution PET scanner to compare two computational methods, one based on a homogeneous tissue model (BFM) and one that accounts for tissue kinetic heterogeneity (SAIF). With high resolution data and good counting statistics, estimates of the variable of interest in our study, rCPS, were equally good with BFM and SAIF; the impact of kinetic heterogeneity on rCPS estimates was negligible. Thus the BFM analysis based on a homogenous tissue model can be used; this is not only simpler, but results in a substantially lower computational cost. When spatial resolution was reduced by smoothing the data, effects of tissue kinetic heterogeneity on estimates of rCPS were notably higher: BFM overestimated rCPS, a result consistent with applying a homogeneous tissue model to kinetically heterogeneous data. In this case, analyses need to take heterogeneity into account, even though this results in higher computational costs.

In the high resolution data set, SAIF identification of kinetic heterogeneity highlights that ~45% of brain voxels should be classified as kinetically heterogeneous in L-[1-^11^C]leucine studies; this violates the assumption of tissue homogeneity usually standard in PET data analyses and, in particular, in the BFM method used here. It is worth noting that the voxels classified as kinetically heterogeneous tended to be located along borders of gray and white matter tissue, where the partial volume effect would be expected to play a stronger role. The biggest effect of neglecting heterogeneity in the model for quantification of rCPS was found in voxels with the lowest values of rCPS and in regions containing a large fraction of low-rCPS voxels. In particular, neglecting heterogeneity had the largest effect on rCPS in regions that contained low-rCPS voxels interspersed with higher-rCPS voxels. The regions with highest differences between SAIF and BFM estimates of rCPS included the amygdala and hippocampus (15% and 17% respectively). Intermediate differences were found in white matter and cerebellum (~6%). For the remaining regions the two methods produced very similar results (absolute relative differences <5%). Interestingly, the injected tracer dose played a role in the differences between the SAIF and BFM estimates of rCPS, with higher injected doses associated with lower differences. This underlines the importance of good counting statistics for PET imaging studies^33^.

When low resolution PET data were generated by smoothing the HRRT images, SAIF estimates of kinetic heterogeneity were found for ~75% of the brain voxels. The increased fraction of heterogeneous voxels is as expected: greater partial volume effects are associated with lower spatial resolution. The distribution of these kinetically heterogeneous voxels was still along the white matter-gray matter borders, but was more extensive. The higher heterogeneity resulted in higher discrepancy between rCPS estimated with the SAIF and BFM methods (~12%), an effect consistent across the tissue ROIs. We note that the effect of injected dose on rCPS estimates was not significant for most of the ROIs as had been found in the analysis of original HRRT data. This could be the result of a lowering of the noise level in the data by the smoothing process.

Findings from the simulation studies were consistent with observations from the analysis of the measured data. The simulations help to identify the conditions in which failure to account for tissue kinetic heterogeneity can lead to biases in estimates of rCPS. In the homogenous tissue simulation scenario, BFM outperformed SAIF despite the fact that both methods support a homogeneous tissue model. SAIF is characterized by more degrees of freedom than BFM and the presence of data noise tends to lead to overfitting and overestimation of tissue kinetic heterogeneity. This behavior is typical of spectral-analysis-based methodologies^23^. In the heterogeneous tissue simulation scenario, BFM underperformed compared to SAIF in terms of bias in rCPS. At high voxel-level noise, however, overall performances in terms of RMSE of BFM and SAIF were equivalent.

Ideally for PET quantification, one would expect better performance for the model whose kinetic assumptions best match with the kinetics of the investigated data. In practice this is not always the case, especially for voxel level analysis, due to its high level of noise. Both simulation and measured data results show that one cannot separate effects of true kinetic heterogeneity in the tissue from effects resulting from the measurement system, including partial volume and noise. SAIF, with its overcomplete representation of the underlying kinetics, provides a method to help find a good trade-off between model kinetic flexibility and robustness to noise.

Some limitations of the study should be taken into account. First, the results reported here refer to the quite unique case of L-[1-^11^C]leucine PET data acquired with the HRRT scanner. Although the applicability of these findings to different tracers, different scanner types or different experimental protocols should be assessed on a case by case basis, the methodology presented here provides a framework for approaching such analyses. Secondly, because of the lack of available L-[1-^11^C]leucine PET data acquired with a scanner other than the HRRT, the low resolution scenario was artificially created by smoothing the high-resolution data. These conditions do not account for the spatial interaction between nearby voxels due to the finite resolution of the HRRT PET scanner on which the data were acquired (FWHM ~2.6 mm). Thirdly, only two out of 8 subjects were injected with a substantially lower dose of tracer (~0.16 mCi/kg), limiting the inferences that can be made concerning the interaction between tracer dose and tissue kinetic heterogeneity; this topic needs further exploration.

The problem of tissue kinetic heterogeneity in PET data quantification is clearly a general problem that goes beyond the measurement of protein synthesis with the L-[1-^11^C]leucine method. Studies have shown that tissue kinetic heterogeneity can be a significant source of bias when PET imaging is used to measure cerebral blood flow, glucose metabolism, or neuroreceptor binding^6–8^. Compared to compartmental modelling, Spectral Analysis-based techniques have the advantage of not requiring that the number of compartments be fixed *a priori*; thus they become applicable to heterogeneous as well as homogeneous tissues without any additional assumptions. Interestingly, measurement of kinetic heterogeneity and its distribution across various tissues has been shown to be an informative parameter linked to the biological “complexity” and can provide useful insight when applied to pathology. In PET oncological studies, for example, tissue kinetic heterogeneity has been shown to provide information about tumor characterization and treatment response^34,35^. In acute lung injury the heterogeneity fraction has been linked to the presence of edema^36^. Further studies are needed to test the reliability of the measure and its biological value in different contexts.

## Conclusion

In this study we showed how voxelwise analysis of L-[1-^11^C]leucine PET data can be affected by the problem of tissue kinetic heterogeneity. The effect on the variable of interest in our study, rCPS, is negligible when we have good counting statistics and high-resolution data, but it becomes significant with poorer spatial resolution. With high-resolution data and poorer counting statistics small biases in rCPS become apparent, but the effects of kinetic heterogeneity and high noise cannot be independently assessed. These findings may be extended to other PET tracers, to tissues other than brain, or to pathological conditions (as tumors or inflammation) that might increase the level of tissue kinetic heterogeneity, but each condition must be assessed on a case by case basis.

## Electronic supplementary material

Supplementary Information
