# Supplementary material for: Impact of tissue kinetic heterogeneity on PET quantification: case study with the L-[1-11C]leucine PET method for cerebral protein synthesis rates
Source: Sci Rep. 2018 Jan 17;8:931. doi: 10.1038/s41598-017-18890-x (PMC5772379; doi:10.1038/s41598-017-18890-x)
Supplement: Supplementary file 1 — Supplementary Information [file 41598_2017_18890_MOESM1_ESM.pdf]

Impact of tissue kinetic heterogeneity on PET quantification: case study with the L-[1-^{11}C]leucine PET method for cerebral protein synthesis rates

Veronese, Bertoldo,Tomasi, Beebe Smith, Schmidt

Basis Function Method

The kinetic model description of total activity in the voxel to be analyzed (Figure 1) is given by

$$C_T^*(t)=(1-V_b)\left\{\left(\frac{K_1(k_2+k_3)}{k_2+k_3+k_4}\right)\int_0^t C_p^*(\tau)e^{-\beta(t-\tau)}d\tau + \left(\frac{K_1k_4}{k_2+k_3+k_4}\right)\int_0^t C_p^*(\tau)d\tau + V_D C_c^*(t)\right\}+V_b C_b^*(t), \quad [1]$$

where $\beta=k_2+k_3+k_4$. Let

$$A(t) = \int_0^t C_p^*(\tau)\, d\tau \quad [2]$$

$$B(\beta,t) = \int_0^t C_p^*(\tau)e^{-\beta(t-\tau)}d\tau \quad [3]$$

$$D(t) = \left[C_b^*(t) - V_D C_c^*(t)\right]. \quad [4]$$

Eq [1] can be rewritten as:

$$C_T^*(t) - V_D C_c^*(t) = \theta_1 A(t) + \theta_2 B(\beta,t) + \theta_3 D(t) \quad [5]$$

where

$$\theta_1=(1-V_b)\frac{K_1k_4}{k_2+k_3+k_4} \quad [6]$$

$$\theta_2=(1-V_b)\frac{K_1(k_2+k_3)}{k_2+k_3+k_4} \quad [7]$$

$$\theta_3=V_b. \quad [8]$$

If the value of β were known, Eq [5] would be linear in the parameter vector $\boldsymbol{\theta}=[\theta_1\ \theta_2\ \theta_3]^T$ which could be quickly estimated using standard weighted linear least squares. The superscript T denotes matrix transpose.

For any fixed value of β, let **a**, **b**(β), **d**, and **y** be the (column) vectors computed from blood and brain measurements evaluated at the n frame times $t_1, t_2, ..., t_n$:

$$\mathbf{a} = [A(t_1) \quad A(t_2) \ \ldots \ A(t_n)\,]^T \qquad [9]$$

$$\mathbf{b}(\beta) = [B(\beta,t_1) \quad B(\beta,t_2)) \ \ldots \ B(\beta,t_n)]^T \qquad [10]$$

$$\mathbf{d} = [D(t_1) \quad D(t_2) \ \ldots \ D(t_n)\,]^T \qquad [11]$$

$$\mathbf{y} = [C_T^*(t_1) - V_D C_c^*(t_1) \quad C_T^*(t_2) - V_D C_c^*(t_2) \ \ldots \ C_T^*(t_n) - V_D C_c^*(t_n)\,]^T \qquad [12]$$

Define the (n x 3) matrix $\mathbf{X}(\beta)$ as

$$\mathbf{X}(\beta) = [\mathbf{a} \ \ \mathbf{b}(\beta) \ \ \mathbf{d}] \qquad [13]$$

and the (n x n) diagonal weighting matrix **W** as

$$\mathbf{W} = \mathrm{diag}\left(1/\,\mathrm{Var}(C_T^*(t_j))\right) \qquad [14]$$

where $\mathrm{Var}(C_T^*(t_j))$ is assumed proportional to $\left[e^{\gamma t_j} C_T^*(t_j)\right]/\Delta t_j$, γ is the decay constant for ^{11}C and Δt_j is the length of Frame j. The weighted least squares estimate for the parameter vector θ for a fixed value of β is

$$\theta = \left[(\mathbf{X}^T \mathbf{W} \mathbf{X})^{-1} \mathbf{X}^T \mathbf{W}\right] \mathbf{y} \qquad [15]$$

and the weighted residual sum of squares is given by

$$\mathbf{WRSS} = \mathbf{y}^T \left[\mathbf{W} - \mathbf{WX} (\mathbf{X}^T \mathbf{W} \mathbf{X})^{-1} \mathbf{X}^T \mathbf{W}\right] \mathbf{y} \qquad [16]$$

where $\mathbf{I}$ is the (n x n) identity matrix. For simplicity, we have dropped the explicit notational dependence of **X** on β, but the dependence remains and must be implicitly

understood. We will be evaluating the equations for $\boldsymbol{\theta}$ and **WRSS** for all voxels in the brain, but we note that if we use the same weights for all voxels, the matrices $\left[(\mathbf{X}^T \mathbf{W} \mathbf{X})^{-1} \mathbf{X}^T \mathbf{W}\right]$ and $\left[\mathbf{W} - \mathbf{W}\mathbf{X} (\mathbf{X}^T \mathbf{W} \mathbf{X})^{-1} \mathbf{X}^T \mathbf{W}\right]$ need be computed only once for each possible value of β.

The idea of BFM is to define a grid of *m* values for β in the physiological range, $\beta_1,\beta_2,...\beta_m$, solve for each the corresponding weighted linear least squares problem, and select the value β^* which gives rise to the smallest WRSS. Let $\boldsymbol{\theta}^*=[\theta_1^* \ \theta_2^* \ \theta_3^*]^T$ be the estimated parameter vector at $\beta=\beta^*$. Eqs [6]-[8] can then be solved to yield values of the rate constants (K_1,k_2+k_3,k_4) and blood volume (V_b) as

$$K_1 = \frac{\theta_1^* + \theta_2^*}{1 - \theta_3^*} \quad [17]$$

$$k_2+k_3 = \frac{\theta_2^* \beta^*}{\theta_1^* + \theta_2^*} \quad [18]$$

$$k_4 = \frac{\theta_1^* \beta^*}{\theta_1^* + \theta_2^*} \quad [19]$$

$$V_b=\theta_3^* \quad [20]$$

Furthermore, λ and rCPS can be expressed as:

$$\lambda=\left(\frac{\theta_1}{\theta_0 + \theta_1}\right) \quad [21]$$

and

$$rCPS = K_1\left[\frac{1-\lambda}{\lambda}\right]C_p . \quad [22]$$

The unconstrained least-squares approach outlined above is the initial step in the Basis Function Method (BFM) of Tomasi et al (*Journal of Cerebral Blood Flow and Metabolism* **29**, 1317-1331, 2009).

Revised Basis Function Method

Estimates of the rate constants and blood volume are not physiologically meaningful unless they satisfy certain conditions, namely $K_1>0$, $k_2+k_3>0$, $k_4\geq0$, $1>V_b\geq0$. It can be readily shown that, given $\beta>0$, this set of conditions is equivalent to constraining $\boldsymbol{\theta}$ such that $\theta_1\geq0$, $\theta_2>0$ and $1>\theta_3\geq0$. In the revised BFM, if the best-fitting parameters $\boldsymbol{\theta}$ estimated with the unconstrained least squares fit do not satisfy the requisite constraints, we enlarge the set of possible solutions as described below.

Let X_i^{123}= [**a** **b**(β_i) **d**] be the X matrix as defined above for $\beta=\beta_i$, let θ_i^{123} be the corresponding least squares estimates, and WRSS_i^{123} the weighted residual sum of squares. Define $\boldsymbol{\theta}^{123}$ as the (3 x m) matrix of least squares estimates and $\mathbf{WRSS}^{123}$ as the m-vector of weighted residual sums of squares for each of the m values of β in the grid. The superscript '123' denotes that columns one through three of **X** were used in determining the least squares solution; it also identifies which parameters were estimated. In this case $\theta_i^{123}=[\theta_1\ \theta_2\ \theta_3]^T$.

Next consider the set of least squares problems, defined for all m values of β, which are constrained so that $\theta_3=V_b=0$. These problems are solved as previously

described, but using $\mathbf{X}_i^{12}$= [$\mathbf{a}$ $\mathbf{b}(\beta_j)$] in the estimation. Denote the (2 x *m*) matrix of the parameter estimates and the *m*-vector of weighted residual sums of squares as $\boldsymbol{\theta}^{12}$ and $\mathbf{WRSS^{12}}$ respectively. Thirdly, consider the set of least squares problems in which θ_1=0 (which implies that k_4=0) by using $\mathbf{X}_i^{23}$= [$\mathbf{b}(\beta_j)$ $\mathbf{d}$] and determining $\boldsymbol{\theta}^{23}$ and $\mathbf{WRSS^{23}}$. Fourthly, we include the case where $\theta_1=\theta_3$=0, which implies both V_b=0 and k_4=0, by using $\mathbf{X}_i^{2}$= [$\mathbf{b}(\beta_j)$] and determining $\boldsymbol{\theta}^{2}$ and $\mathbf{WRSS^{2}}$.

Due to the use of constraints on θ in all cases except the first, we have the relationships that $\mathbf{WRSS^{123} \leq WRSS^{12} \leq WRSS^{2}}$ and $\mathbf{WRSS^{123} \leq WRSS^{23} \leq WRSS^{2}}$, as a constrained solution cannot fit better than an unconstrained one. Of course many of the least squares estimates in these solution sets may not satisfy all constraints on θ. Thus our final step is to find the value β^* which gives rise to the smallest WRSS over the subset of values of {$\mathbf{WRSS^{123}, WRSS^{12}, WRSS^{23}, WRSS^{2}}$} in which the corresponding least squares estimates meet all constraints on θ. If there is no such value of β in any of the problem sets where the corresponding least squares estimate meets all constraints, then there is no solution and the voxel must be discarded. Otherwise, the rate constants (K_1,k_2+k_3,k_4), blood volume (V_b), λ, and rCPS can be determined from Eqs [17] through [22]. In practice we have not encountered a failure to find a solution.
